# National prevalence of diarrhea and associated factors among children under five in Afghanistan

**DOI:** 10.21203/rs.3.rs-9006888/v1

**Published:** 2026-04-11

**Authors:** Mohammad Omer Malikzai, Ghulam Farooq Mansoor, Said Iftekhar Sadaat, Ali Mirzazadeh

**Affiliations:** Cure International Hospital. Kabul; Health Protection and Research Organization. Kabul; University of California San Francisco. California. San Francisco; University of California San Francisco. California. San Francisco

**Keywords:** Afghanistan, Diarrhea, Children under five years, Determinants of diarrhea, Multiple Indicators Cluster Survey

## Abstract

**Background:**

Diarrhea is a leading cause of mortality and morbidity among children under five in Afghanistan. Assessing the prevalence and identifying its key determinants is essential for reducing the disease burden and effective public health interventions.

**Methods:**

The study analyzes prevalence and determinants of diarrhea among children under five years, using secondary data from Multiple Indicator Cluster Survey (MICS) in Afghanistan 2022–2023, including 32,989 children representing all 34 provinces, including urban and rural regions. Multivariable logistic regression was performed to calculate adjusted odds ratios (AOR) for the association between covariates and the diarrhea outcome.

**Results:**

Overall, 38.2% of children had diarrhea (95% CI: 37.0–39.4). After adjustment, children aged 6–11 months (AOR = 1.80, p < 0.001), 12–23 months (AOR = 1.72, p < 0.001), 24–35 months (AOR = 1.55, p < 0.001), and 36–47 months (AOR = 1.16, p < 0.001) had significantly higher odds of diarrhea compared with infants aged 0–5 months. Children of mothers aged 20–29 years (AOR = 0.78, p < 0.01) and 30–39 years (AOR = 0.77, p < 0.01) had significantly lower odds of diarrhea compared with those born to mothers aged < 20 years. Children from the richest households had significantly lower odds of diarrhea (AOR = 0.60, p < 0.01), as did those whose mothers had above-primary education (AOR = 0.80, p < 0.01). Higher odds of diarrhea observed among children living in households practicing open defecation (AOR = 1.19, p < 0.01), and in the South (AOR = 1.61, p < 0.001), and East (AOR = 1.47, p < 0.001) regions compared to Central region.

**Conclusion:**

More than one in three children had diarrhea. Childhood diarrhea in Afghanistan remains driven by modifiable factors, particularly unsafe weaning-period practices, low maternal education, and poor sanitation, with children aged 6–35 months and those living in households practicing open defecation at substantially higher risk. Strengthening infant and young child feeding interventions, expanding girls’ education, maternal health literacy, accelerating sanitation and behavior change programs could substantially reduce diarrhea burden. Targeted implementation in high-risk regions, especially the South and East, is essential to address persistent geographic inequities.

## Background

Diarrhea among children under five years of age is a global public health concern and a major cause of mortality and morbidity in this age group. Globally, diarrhea accounted for approximately 9% of deaths among children under five years of age in 2021, which was 1,200 deaths per day or about 444,000 deaths per year [1].

The burden of diarrhea among children under five years of age varies in different regions. Among the Asian countries, Afghanistan has remained having the highest prevalence of diarrhea among children under five years of age. The Afghanistan Demographic and Health Survey (DHS) in 2015 reported that 26.2% of the under-five children had diarrhea in the two weeks preceding the survey [2]. In Pakistan, the Multiple Indicator Cluster Survey (MICS) 2017–18, a survey of 38,405 households, reported that 13.7% of children under five years of age had diarrhea [3]. While India’s National Family Health Survey 2019–20 reported that 7.3% of children under five years of age had diarrhea, two weeks preceding the survey [4].

Many key factors influence the prevalence of diarrhea among children under five years of age. For example, a study done in Punjab, Pakistan, shows an inverse association of maternal education level and prevalence of diarrhea. The prevalence of diarrhea among children, of mothers with no formal schooling, was the highest (15.21%), compared with the lowest prevalence of (10.45%) in children whose mothers had obtained higher education [3]. Similar results have been shown from a study done in Yemeni children, where the children with uneducated mothers and caregivers have a 49% higher likelihood of diarrhea prevalence. [5] The findings highlight the significance of maternal education and the prevention of diarrhea.

The prevalence and factors that influence the burden of diarrhea among children have not been thoroughly investigated in recent times in Afghanistan. The previous population level study in Afghanistan was conducted in 2018 and 2015. It is relevant to measure the prevalence of diarrhea and its associated factors in a study conducted 5 years later in 2022/23, particularly at a crucial juncture when a change in the regime has occurred.

This paper aims to present the prevalence of diarrhea among children under five years of age and relevant factors, including demographic, parental, and household factors. We hope the findings will help the health system take preventative measures for diarrheal disease in the population under five and build national strategies for better health and education of our people.

## Methods

### Source of data and sampling design

We performed secondary analysis of the Afghanistan MICS, which was conducted in 2022–23 by UNICEF Afghanistan in collaboration with the Afghanistan National Statistics and Information Authority (NSIA). The MICS survey, approved by the NSIA Technical Committee, was a cross-sectional survey using a two-stage stratified cluster sampling design. In the first stage, the clusters/enumeration areas were randomly selected from a sampling frame of approximately 30,000 enumeration areas in 34 provinces. In the second stage, the households were systematically selected based on an already conducted household listing operation. The original survey database comprised 23,213 households across 34 provinces, with 33,398 eligible children, for whom mothers or caregivers of 32,989 children were available for interview within the selected households. The interviews were administered using a standard questionnaire. Verbal consent was taken for adult participants, including caretakers of children aged less than 5 years. Each respondent was assured of the confidentiality of their information. It was also ensured that the participation was voluntary.

### Ethics approval and consent to participate

Our study was based on secondary data analysis of publicly available cross-sectional data from Afghanistan Multiple Indicator Cluster Survey (MICS) by UNICEF. Where the survey was conducted in accordance principles with Declaration of Helsinki, with all identifying information removed; therefore. The original study protocol was reviewed and approved by a technical committee of the NSIA.

#### Funding:

The authors received no funding for the work.

The study is from anonymized MICs survey, where initial informed consent was obtained by the survey teams. No additional ethical approval is required to this study.

#### Availability of data and materials:

https://mics.unicef.org/surveys?display=card&f%5b0%5d=region:2521&f%5b1%5d=datatype:0&f%5b2%5d=year:2023&f%5b3%5d=year:2022

### Variables and statistical analyses

The outcome variable “Diarrhea” was derived from the question “Did your child have diarrhea in the past two weeks?”. The independent variables were age of child, gender of child, residence (urban/rural), household size, mothers’ and fathers’ education level, mothers’ age, location of the household in one of the 8 regions of the country, the household wealth, source of drinking water, and sanitation. We used child sampling weights that were produced by the MICS survey team; we applied the Svy command in Stata 18 (Stata Corporation, College Station, Texas, US) to control for selection probability in different provinces and produced weighted results. We grouped the 34 provinces into 8 regions that are geographically and culturally different from each other. We ran the frequencies and percentages of the sample characteristics. We also measured the distribution of the outcome variable (i.e., diarrhea) across various potential predictors using the chi2 test and producing p-values. Bi-variable logistic regression analyses were performed to measure the association of diarrhea with the potential predictors and produced crude Odds Ratios. Predictor variables for which the results were significant at <0.05 in bivariable analysis were put into the multivariable logistic regression model, producing adjusted Odds Ratios.

## Results

### Sample characteristics

A total of 32,989 children under the age of five were included in the Afghanistan MICS 2022–23 survey, and caretakers were interviewed. The largest proportion (21.4%) of children were in the age group 48–59 months, followed by 36–47 months (20.2%) and 12–23 months (19.4%). The majority of children resided in rural areas (77.3%). The sex distribution was nearly balanced, with males comprising 51.2%.

Maternal education levels were low: 78.8% of mothers had no formal education, 9.5% had attained primary education, and 11.8% had completed education beyond the primary level. Maternal age was concentrated in the 20–29 year range (51.0%), followed by 30–39 years (36.0%). Father’s education showed greater variation, with 51.6% having no formal education, 12.6% completed primary education, and 27.9% attained education beyond the primary level. In 7.8% of cases, the biological father was not present in the household. The wealth index distribution was relatively even, though the poorest quintile accounted for 21.9% of the sample. Household size was notably large, with 36.8% of children living in households with 11 or more members and 28.7% living in households with 8–10 members. Regional representation varied, with the Central (17.4%), South (16.8%), and North (15.4%) regions comprising the largest shares of the population of children under five years of age. The Central Highlands accounted for the smallest proportion (3.1%). Regarding access to basic services, 73.2% of children lived in households with improved sources of drinking water. Improved sanitation facilities were available to the households where 55.1% of the children resided, while 21.4% resided in households with no sanitation facility, practicing open defecation. Breastfeeding prevalence among children aged 0–23 months was high, with 88.7% having ever been breastfed.

### Prevalence of Diarrhea in children by various characteristics

Overall, 38.2% of children had diarrhea (95% CI: 37.0–39.4). Prevalence varied significantly by age (p<0.001), peaking during the weaning period: 45.9% at 6–11 months and 44.4% at 12–23 months, remaining elevated at 24–35 months (42.3%), and lower at 0–5 months (33.0%) and 48–59 months (30.5%). Rural children had a higher prevalence than urban (39.6% vs. 33.5%; p<0.001). There was no sex difference (male 38.7% vs. female 37.6%; p=0.156).

Children of mothers with above-primary education had the lowest prevalence (28.7%) compared with mothers with no education (39.4%) or primary education (39.4%) (p<0.001). A similar pattern was seen for father’s education (p<0.001): above-primary 34.9% vs. no education 39.5% and primary 40.5%; children whose biological father was not in the household, 37.1% had diarrhea.

By mother’s age, prevalence of diarrhea in children differed significantly (p=0.001). Children of the youngest mothers (15–19 years) had the highest prevalence of diarrhea at 46.7%. Prevalence was lower for children with mothers aged 20–29 years, 38.0% and 30–39 years, 37.5%. It rose slightly among children whose mothers were aged 40–49 years at 39.8%.

By wealth, prevalence of diarrhea was highest, 41.6% in the poorest quintile and lowest 28.6% in the richest quintile, showing a clear gradient (p<0.001). Household size was not associated with diarrhea in children (p=0.666). Marked regional heterogeneity in the prevalence of diarrhea among children was evident (p<0.001), with the Southern region (49.8%), Eastern region (46.3%), the highest, and Central region (33.6%) North Eastern region (33.5%), and Western region (32.8%), had the lowest prevalence of diarrhea among children less than five years of age.

Water source (improved vs. unimproved) showed no association with diarrhea among children less than 5 years (38.5% vs. 37.3%; p=0.372). In contrast, use of sanitation facility was strongly associated with diarrhea (p<0.001): the prevalence of diarrhea among children in the households with improved sanitation facilities was 34.6%, with unimproved facilities was 39.5%, and open defecation (no sanitation facility) had the highest prevalence of diarrhea at 45.9%. Among children 0–23 months, those never breastfed had a higher prevalence of diarrhea than those ever breastfed (47.1% vs. 41.3%; p<0.001).

### Factors associated with the prevalence of diarrhea in children

Table 3 presents the analysis that examined factors associated with diarrhea among children under five years of age using logistic regression analysis. After adjustment, children aged 6–11 months (AOR = 1.80, p < 0.001), 12–23 months (AOR = 1.72, p < 0.001), 24–35 months (AOR = 1.55, p < 0.001), and 36–47 months (AOR = 1.16, p < 0.001) had significantly higher odds of diarrhea compared with infants aged 0–5 months. Children of mothers aged 20–29 years (AOR = 0.78, p < 0.01) and 30–39 years (AOR = 0.77, p < 0.01) had significantly lower odds of diarrhea compared with those born to mothers aged <20 years. Children from the richest households had significantly lower odds of diarrhea (AOR = 0.60, p < 0.01), as did those whose mothers had above-primary education (AOR = 0.80, p < 0.01). Higher odds of diarrhea observed among children living in households practicing open defecation (AOR = 1.19, p < 0.01), and in the South (AOR = 1.61, p < 0.001), and East (AOR = 1.47, p < 0.001) regions compared with the Central region.

## Discussion

This study analyzed nationally representative data from the Afghanistan MICS 2022, revealing 38.2% prevalence of diarrhea among children under five in the two weeks preceding the survey. This is notably higher than findings from the previous national surveys: 26.2% in the DHS 2015 and 18.1% in the AHS 2018.[6, 7]. This increase may reflect the deterioration of the situation, due to the contraction of foreign assistance to Afghanistan’s social sector in recent years, with support now largely restricted to humanitarian aid.

The multivariable analysis highlighted several significant predictors of diarrhea, including child age, maternal education and age, paternal education, household wealth, sanitation practices (particularly open defecation), and residence in the South and East regions. In contrast, breastfeeding status, drinking water source, child sex, household size, and urban/rural residence were not significantly associated after adjustment.

We found several child-related factors associated with higher risk of diarrhea. The results showed that Children aged 6–35 months exhibited significantly higher odds of diarrhea compared to infants aged 0–5 months. The strong association between child age and diarrhea prevalence is consistent with global patterns, where the risk peaks during the weaning period and early toddlerhood. This vulnerability is likely due to increased exposure to contaminated food, water, and environmental pathogens as children begin complementary feeding and mobility increases. Similar age-related trends have been reported in prior analyses of the Afghanistan Demographic and Health Survey (AfDHS) 2015 and in other low-resource settings such as Ethiopia and Bangladesh, underscoring the need for age-targeted hygiene and nutrition interventions.[8, 9]

We also found several important maternal factors for diarrhea.Maternal education above primary emerged as protective, echoing findings from Afghanistan’s 2015 DHS and consistent with evidence across contexts: Studies in Pakistan, Yemen, and India revealed that maternal education is inversely associated with the prevalence of diarrhea in children (3, 5,9,10). In Ethiopia, children of mothers with secondary or higher education had significantly lower odds of diarrhea (AOR ≈ 0.78) compared to those whose mothers had no schooling[10]. Educated mothers are more likely to adopt hygiene practices, recognize early signs of illness, and seek timely care, which reduces both the incidence and severity of diarrhea. Interestingly, children of mothers with only primary education had slightly elevated odds of diarrhea, suggesting that partial education may not be sufficient to influence health behaviors; this is an in-depth insight echoed in prior qualitative research. [11, 12] The protective association between maternal age and diarrhea among children under five in Afghanistan reflects broader patterns observed in low-resource settings. In our analysis, children of mothers aged 20–39 years had significantly lower odds of diarrhea compared to those of below 20 years’ age. This finding is supported by a secondary analysis of the Afghanistan Demographic and Health Survey (AfDHS) 2015, which found that younger maternal age was significantly associated with higher diarrhea prevalence among children, even after adjusting for sanitation and socioeconomic factors.[2]. Globally, similar trends have been documented. For example, Rutstein (2005) demonstrated across multiple DHS datasets that maternal age below 20 years is consistently associated with poorer child health outcomes, including increased risk of diarrhea.[13]. Younger mothers may lack experience, autonomy, and access to health information, which can compromise child hygiene and feeding practices.

Regarding the household level factors, we found household wealth, and lack of access to sanitation (i.e, open defecation practice) are strongly associated with risk of diarrhea. Household wealth showed an inverse gradient, children in wealthier quintiles were less likely to experience diarrhea, reflecting global findings where poverty elevates risk via poor sanitation, overcrowding, and limited health service access. Families evaluated in Punjab, Pakistan, having a higher income had lower diarrhea prevalence, followed by middle-income families. Regarding sanitation, open defecation was significantly associated with higher diarrheal risk. It also showed a higher incidence of diarrhea among children having no toilet facility 19.3%, compared with those having toilets 11.4%, while children practicing open defecation in rural areas of India had and AOR: 1.35(95% CI: 1.28–1.42) for diarrhea.[3, 14] In a study from Ethiopia, under-five children in open defecation households had a diarrhea prevalence of 38% vs 26% in open-defecation-free households[15].

We also observed significant regional disparities. The South and the East regions showed a higher risk, while the West showed a lower risk. Further exploring the data reveals that the higher risk in the South and East coincides with multiple vulnerabilities, including higher open defecation, larger household sizes, lower breastfeeding, and lower maternal education. Tailored regional strategies are essential to address these contextual vulnerabilities.

The main strength of this study is the use of nationally representative MICS 2022 data, allowing robust and generalizable findings. However, the study is limited by its cross-sectional design, which prevents causal inference, and reliance on maternal recall of diarrhea in the past two weeks, which may introduce recall or reporting bias.

Despite these limitations, we provided an updated national and subnational prevalence of prevalence diarrhea. More than one in three children had diarrhea. Childhood diarrhea in Afghanistan remains driven by modifiable factors, particularly unsafe weaning-period practices, low maternal education, and poor sanitation, with children aged 6–35 months and those living in households practicing open defecation at substantially higher risk. Strengthening infant and young child feeding interventions, expanding girls’ education and maternal health literacy, and accelerating sanitation and behavior change programs could substantially reduce diarrhea burden. Targeted implementation in high-risk regions, especially the South and East, is essential to address persistent geographic inequities. Given the high burden of diarrhea, these interventions remain essential to achieving reductions in under-five morbidity and mortality and to advancing Afghanistan’s progress toward SDG targets.

## Figures and Tables

**Table 1:** Background characteristics of the sample children 0–59 months, MICS 2022–23 Afghanistan (N=32989)

Variables	n	Weighted %
	**32989**	100.0
**Children Age group**		
0–5 months	3,437	10.4
6–11 months	3,167	9.7
12–23 months	6,177	19.4
24–35 months	6,342	18.9
36–47 months	6,826	20.2
48–59 months	7,040	21.4
**Residential Location**		
Urban	4,991	22.6
Rural	27,998	77.3
**Sex of the child**		
Male	16,841	51.2
Female	16,148	48.7
**Mothers' Education**		
No education	27,293	78.8
Primary education	2,491	9.5
Above primary education	3,205	11.8
**Mother’s age (N)**	**32409**	
15–19	944	3.3
20–29	16,256	51.0
30–39	11,737	36.0
40–49	3,472	10.7
**Father’s education (N)**	**32989**	
No education	18004	51.6
Primary education	3557	12.6
Above primary education	9018	27.9
Biological father not in HH	2410	7.8
**Wealth Index (N)**	**32989**	
Poorest	7,824	21.9
Second	7,823	21.3
Middle	7,302	20.2
Fourth	5,976	19.3
Richest	4,064	17.2
**Household size (N)**	**32989**	
2–5 members	4,223	14.6
6–7 members	6,112	19.7
8–10 members	9,499	28.7
11+ members	13,155	36.8
**Regions (N)**	**32989**	
Central region	5,496	17.4
West Region	3,519	14.3
South Region	5,606	16.8
East Region	4,469	10.5
North Region	4,561	15.4
North East	3,349	13.0
South East	4,485	9.0
Central highlands	1,504	3.1
**Source of drinking water (N)**	**32989**	
Improved	22,198	73.2
Unimproved	10,791	26.8
**Sanitation facility (N)**	**32989**	
Improved	16,585	55.1
Unimproved	8,880	23.4
open defecation	7,524	21.4
**Ever Breastfed 0–23 months (N)**	**18963**	
Yes	16,510	88.7
No	2,453	11.2

**Table 2:** Prevalence of Diarrhea among children 0–59 months by sociodemographic characteristics, MICS 2022–23 Afghanistan (N=32989)

Variables	% weighted	95% CI
Diarrhea Overall	38.2	37.0–39.4
**By children's age group**	P<0.001	
0–5 months	33.0	30.6–35.5
6–11 months	45.9	43.1–48.8
12–23 months	44.4	42.5–46.2
24–35 months	42.3	40.4–44.2
36–47 months	35.5	33.6–37.4
48–59 months	30.5	29.5–32.2
**By Residential Location**	<0.001	
Urban	33.5	30.5–36.5
Rural	39.5	38.2–41.0
**By the Sex of the child**	P= 0.156	
Male	38.7	37.2–40.2
Female	37.6	36.3–39.0
**By Mothers' Education**	P <0.001	
No education	39.4	38.1–40.8
primary education	39.4	36.0–42.9
above primary education	28.7	26.3–31.2
**By Mother's age**	0.001	
15–19	46.7	42.4–51.0
20–29	38.0	36.5–39.4
30–39	37.5	35.7–39.4
40–49	39.8	37.3–42.5
**By Father's education**	P<0.001	
No education	39.5	38.1–41.0
Primary education	40.5	37.9–43.2
above primary education	34.9	33.0–36.9
Biological father not in HH	37.1	34.2–40.1
**By Wealth Index**	P<0.001	
Poorest	41.6	39.4–43.8
second	40.4	38.4–42.5
Middle	41.4	39.4–43.3
Fourth	37.1	34.9–39.3
Richest	28.6	25.9–31.5
**By household size**	P=0.667	
2–5 members	37.0	34.7–39.2
6–7 members	38.7	36.8–40.7
8–10 members	38.3	36.5–40.1
11+ members	38.3	36.5–40.1
**By Regions**	P<0.001	
Central region	33.6	30.8–36.5
West Region	32.8	29.3–36.5
South Region	49.8	46.9–52.7
East Region	46.3	42.3–50.4
North Region	34.9	31.8–38.2
North East	33.5	30.9–36.3
South East	38.0	33.6–42.6
Central highlands	34.8	29.7–40.3
**By Source of drinking water**	P=0.372	
Improved	38.5	37.0–40.0
Unimproved	37.3	35.1–39.5
**By the type of sanitation facility**	P<0.001	
Improved	34.6	33.1–36.2
Unimproved	39.5	37.7–41.3
Open defecation	45.9	43.5–48.4
**By the child’s breastfeeding status (0–23 months)**	P<0.001	
Yes	41.3	40.0–42.7
No	47.1	44.1–50.1

**Table 3:** Factors associated with the prevalence of diarrhea among children 0–59 months, MICS 2022–23 Afghanistan (N=32989)

Variables	Diarrhea in Children <5 years	
	OR (95% CI)	AOR (95% CI)
**Children Age Group (Reference category 0–5 months)**		
0–5 months	Baseline	Baseline
6–11 months	1.72(1.48–2.01)**	1.80(1.54–2.1)**
12–23 months	1.62(1.43–1.83)**	1.72(1.52–1.95)**
24–35 months	1.49(1.31–1.69)**	1.55(1.35–1.77)**
36–47 months	1.12(0.98–1.27)	1.16(1.02–1.33)*
48–59 months	0.89(0.78–1.01)	0.91(0.80–1.04)
**Location (Reference category urban)**		
Urban	Baseline	Baseline
Rural	1.3(1.11–1.52)**	0.93(0.79–1.09)
**Sex of child (Reference category male)**		
Male	Baseline	Baseline
Female	0.96(0.9–1.02)	
**Mothers' Education (Reference category: No education)**		
No education	Baseline	Baseline
Primary	1(0.86–1.16)	1.18(1.02–1.36)*
Above primary	0.62(0.55–0.7)**	0.8(0.7–0.92)**
**Mother’s age (Reference category <20 years)**		
15–19	Baseline	Baseline
20–29	0.7(0.59–0.83)**	0.78(0.65–0.93)**
30–39	0.69(0.57–0.82)**	0.77(0.63–0.93)**
40–49	0.76(0.62–0.93)**	0.83(0.67–1.02)
**Father's education (Reference category No Education)**		
No education	Baseline	Baseline
Primary	1.04(0.93–1.17)	1.16(1.02–1.3)*
Above primary	0.82(0.75–0.9)**	1.02(0.92–1.12)
Biological father not in HH	0.9(0.79–1.03)	1.07(0.93–1.22)
**Wealth Index (Reference category Poor)**		
Poorest	Baseline	Baseline
Second	0.96(0.85–1.07)	0.94(0.85–1.05)
Middle	0.99(0.88–1.12)	0.97(0.86–1.1)
Fourth	0.83(0.73–0.95)**	0.85(0.73–0.98)*
Richest	0.56(0.48–0.67)**	0.6(0.49–0.72)**
**Regions (reference Central region)**		
Central region	Baseline	Baseline
West Region	0.96(0.78–1.19)	0.8(0.65–0.98)*
South Region	1.96(1.65–2.33)**	1.6(1.35–1.91)**
East Region	1.71(1.39–2.1)**	1.47(1.2–1.81)**
North Region	1.06(0.88–1.28)	0.97(0.8–1.17)
North East	1(0.84–1.19)	0.87(0.73–1.03)
South East	1.21(0.96–1.52)	1.07(0.85–1.33)
Central highlands	1.06(0.81–1.38)	0.86(0.67–1.11)
**“household size (Reference 2–5 members)**		
2–5 members	Baseline	Baseline
6–7 members	1.08(0.96–1.21)	
8–10 members	1.06(0.94–1.19)	
11+ members	1.06(0.94–1.19)	
**Source of drinking water (Reference: Improved source)**		
Improved	Baseline	Baseline
Unimproved	0.95(0.85–1.06)	
**Sanitation facility (Reference: Improved facility)**		
Improved	Baseline	Baseline
Unimproved	1.23(1.12–1.35)**	1.00(0.90–1.11)
Open defecation	1.60(1.42–1.81)**	1.19(1.06–1.35)**
**Ever Breastfed 0–23 months (Reference: yes)**		
Yes	Baseline	Baseline
No	1.26(1.12–1.42)**	1.1(0.97–1.25)
